# Therapeutic advances in the targeting of ROR1 in hematological cancers

**DOI:** 10.1038/s41420-024-02239-1

**Published:** 2024-11-17

**Authors:** Adrian-Bogdan Tigu, Raluca Munteanu, Cristian Moldovan, Drula Rares, David Kegyes, Radu Tomai, Vlad Moisoiu, Gabriel Ghiaur, Ciprian Tomuleasa, Hermann Einsele, Diana Gulei, Carlo M. Croce

**Affiliations:** 1 Medfuture Research Center for Advanced Medicine, Iuliu Hatieganu University of Medicine and Pharmacy Cluj Napoca, Cluj Napoca, Romania; 2 Department of Translational Molecular Pathology, The University of Texas MD Anderson Cancer Center, Houston, TX USA; 3 Department of Neurology, University Hospital and University of Zurich, Zurich, Switzerland; 4 Division of Hematological Malignancies, Sidney Kimmel Comprehensive Cancer Center, Johns Hopkins University, Baltimore, MD USA; 5 Department of Medicine, University of Würzburg, Würzburg, Germany; 6 Department of Cancer Biology and Genetics, The Ohio State University, Columbus, OH USA

**Keywords:** Receptor pharmacology, Lymphopoiesis

## Abstract

Receptor tyrosine kinases (RTKs) are key cell surface receptors involved in cell communication and signal transduction, with great importance in cell growth, differentiation, survival, and metabolism. Dysregulation of RTKs, such as EGFR, VEGFR, HER2 or ROR, could lead to various diseases, particularly cancers. ROR1 has emerged as a promising target in hematological malignancies. The development of ROR1 targeted therapies is continuously growing leading to remarkable novel therapeutical approaches using mAbs, antibody-drug conjugates, several small molecules or CAR T cells which have shown encouraging preclinical results. In the hematological field, mAbs, small molecules, BiTEs or CAR T cell therapies displayed promising outcomes with the clinical trials data encouraging the use of anti-ROR1 therapies. This paper aims to offer a comprehensive analysis of the current landscape of ROR1-targeted therapies in hematological malignancies marking the innovative approaches with promising preclinical and clinical. Offering a better understanding of structural and functional aspects of ROR1 could lead to new perspectives in targeting a wide spectrum of malignancies.

## Facts

- ROR1 expression is directly related to developmental processes during embryogenesis, less expressed in benign adult tissues and blooms in malignancies.
- WNT/ROR1 pathway is linked to tumor progression, resistance to therapy and cell proliferation.
- ROR1 targeted therapy is considered a promising antitumor strategy due to the overexpression of ROR1 on the surface of tumor cells.
- CAR T cell therapy and the use of BiTEs in hematological malignancies broaden the immunotherapies options.

## Questions

- Could ROR1 be co-targeted implying different drug formulations to overcome drug resistance?
- Can CAR T cells be knights in the fight against cancer and enhance the synergistic effect of combinatorial therapies?
- Can anti ROR1 therapies be enhanced by micro RNAs via their interaction with ROR1 and the downstream signaling pathways?

## Introduction

Receptor Tyrosine Kinase-like Orphan Receptor 1 (ROR1) is highly expressed during embryonic development and is involved in cell differentiation, which leads to the formation of, functional tissues and organs. ROR1’s activity is regulated during specific stages of embryogenesis, supporting proper tissue development and cell positioning [1–3].

In adult tissues the expression of ROR1 is downregulated, being almost absent in some tissues. This downregulation is important, as the overexpression in adult tissues is associated with pathological conditions, such as malignancies. Malignant cells use ROR1 expression to promote survival, proliferation and migration, processes normally active in embryonic cells, but redirected by cancer cells to support tumor growth and metastasis. ROR1 supports tumor cell survival by inhibiting apoptosis, while its overexpression drives uncontrolled proliferantion [4–6]. ROR1 overexpression was observed in chronic lymphocytic leukemia (CLL) which is the most studied malignancy in the context of ROR1 targeted therapies [7, 8], as well as in triple-negative breast cancer (TNBC) and other aggressive breast cancer subtypes and non-small-cell lung cancer (NSCLC). Its variable expression across other malignancies makes it a promising target in cancer therapy [9–11].

When ROR1 was first discovered in 1992, it was classified as an orphan receptor due to the absence of an identified ligand. Nowadays it is well established that WNT ligands bind to ROR1, activating downstream signaling pathways. The WNT/ROR1 signaling pathway axis is crucial for tumor progression, resistance to therapy and cell proliferation, driving malignant behaviors in cancer cells. The tumor cell proliferation is triggered via AKT/PI3K pathway, inhibiting apoptosis and supporting the rapid growth of cancer cells. ROR1 signaling also influences MAPK/ERK pathway and promotes survival, while activation of NF-κB pathway contributes to therapy resistance Moreover, ROR1 signaling can indirectly modulate the JAK/STAT pathway, further enhancing tumor cells survival and resistance to therapy [12–15].

Due to ROR’s involvement in various malignant processes, it has become a promising target for treatment. Its limited expression in normal adult tissues reduces the risk of off-target effects, making ROR1-targeted therapies promising with potential of reducing systemic toxicity. Several therapeutic strategies are under development, including monoclonal antibodies, antibody-drug conjugates and chimeric antigen receptor (CAR) T cell therapies. Cirmtuzumab, a well-known monoclonal antibody targeting ROR1 has shown promising results in preclinical and clinical studies. By blocking ROR1 signaling, these therapeutical approaches can disrupt critical pathways that are essential for tumor cell development and survival [4, 16].

CAR T cell therapies targeting ROR1 are also gaining interest in tumors that overexpress ROR1, such as leukemias, lymphomas and solid tumors. Preclinical studies have demonstrated that CAR T cells targeting ROR1 are effective in eliminating tumor cells in vitro and animal models, with early-phase clinical trials assessing their safety and efficacy in humans [17]. Bi-specific T cell engagers (BiTEs) targeting ROR1 are designed to bind both CD3 on T cells and ROR1 on tumor cells, using the immune system’s specificity like CAR T cell therapies [18, 19].

In hematological malignancies, the use of CAR T cell therapies and BiTEs targeting ROR1 is an emerging research area. Preclinical studies suggest that ROR-1 targeted therapies like the use of CAR T and BiTEs, could be effective in treating CLL and Mantle cell lymphoma (MCL) (NCT04763083) [16]. These two approaches can remodel targeted therapies even though ROR1 targeted therapies are not yet widely available in clinical practice.

The aim of this review is to emphasize the need for further development in ROR1 targeted therapies. It synthetizes the existing literature, and gathers results on ROR1-targeted therapies, representing a significant step forward in expanding immunotherapies for hematological malignancies.

## Receptor tyrosine kinase structure and ROR1 signaling pathways

Receptor tyrosine kinases (RTKs) are subtypes of tyrosine kinases that mediate cell communication and regulate biological processes, including cell growth, differentiation, and metabolism [20]. More than fifty RTKs have been identified, most of which have similar protein structures, including an extracellular ligand binding domain, a single transmembrane helix, an intracellular domain which contains a juxtamembrane regulatory region, a tyrosine kinase domain and a carboxyl tail.

**Fig. 1:**
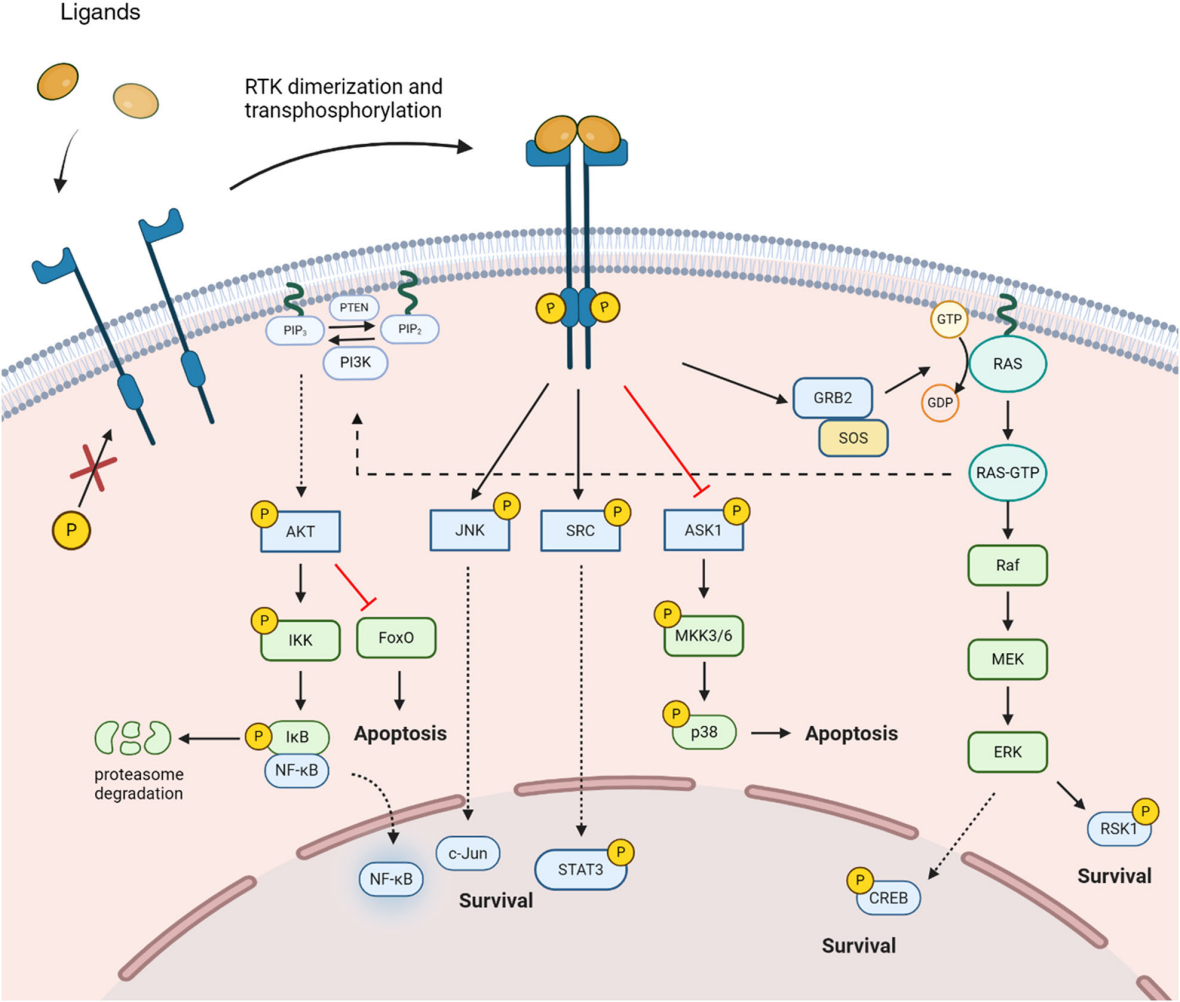
RTKs involvement in cell survival. Ligand-induced receptor dimerization allows the transphosphorilation of the intracellular tyrosine kinase domain, which leads to the activation of multiple pathways. GRB2 is recruited and subsequently binds and recruits SOS, which facilitates the exchange of GDP for GTP on RAS. RAS-GTP, the active form of RAS, initiates downstream signaling via /Raf/MEK/ERK pathway, promoting survival. RAS-GTP activates the PI3K/AKT pathway, leading to IKK tagging IκB for proteasomal degradation, which allows NF-κB to translocate to the nucleus and promote the expression of survival genes. On the other hand, AKT can inhibit FoxO, limiting apoptosis. Moreover, JNK and SRC are activated through receptor phosphorylation, promoting cell survival by enhancing the expression of survival genes like c-Jun and activating transcription factors such as STAT3. The activation of RTK also inhibits ASK1, preventing the activation of its downstream effector p38, which would otherwise promote apoptosis. (Created with BioRender.com).

Most of the RTKs are monomers in the cell membrane and form dimers when the ligand is binding to the extracellular binding domain, which induces autophosphorylation and activates downstream signaling pathways. This dimerization leads to the activation of key proteins involved in cell survival, proliferation and differentiation, as presented in Fig. 1 [21, 22]. Frequent dysregulation and genomic alterations of RTKs, particularly HER2, EGFR, and MET, are involved in the development of various benign conditions and several forms of malignant tumors [21–23].

ROR1, is located on the surface of cells and is typically not expressed in most normal tissues, making it a promising target for CAR T cell and antibody-based therapies. The presence of ROR1 may influence tumorigenesis, as it was shown that ROR1 overexpression can enhance TCL1-driven tumorigenicity in B cell leukemia [24].

Most human CLLs overexpress BCL2 and ROR1. Venetoclax, and cirmtuzumab, an anti BCL2 specific drug, respectively, an anti ROR1 monoclonal antibody, present a synergistic effect against leukemic cells [25]. Thus, ROR1 appears to be a nearly ideal target in the treatment of tumors that overexpress it, that include solid tumors like small cell carcinoma of the lung and intestinal tumors, and hematopoietic tumors such CLL, mantle cell lymphoma (MCL), diffuse large B cell lymphoma (DLBCL) and AML [16, 26–29] The combination of a small molecule ROR1 inhibitor with venetoclax has shown synergistic effects in treating tumor cells, suggesting the potential of ROR1 inhibitors as part of combination therapies for cancer treatment [30].

The increasing importance of RTKs in various cellular processes has led to the identification and characterization of new members within RTK families. Notably, ROR1 and ROR2 were initially recognized in 1992 as two distinct RTKs, with significant amino acid homology to the tyrosine kinase domain of the receptors [31]. The lack of a characterized activator binding molecule determined their name as Receptor Tyrosine Kinase-like Orphan Receptor (ROR), an aspect which is still under investigation to this day.

ROR1 exhibits remarkable sequence conservation across species, underscoring its evolutionary significance. Comparative analysis reveals a 97% homology in the amino acid sequences of ROR1 between mice and humans, indicating that the protein remained largely unchanged during evolution. This high level of conservation suggests that ROR1 has a fundamental role in key biological processes, influencing cell signaling, differentiation and development [32, 33].

Human ROR1 has an immunoglobulin like domain (IG), two CRDs – Frizzled (FZD) and Kringle (KRD) domain on the extracellular region, while the intracellular domains are the tyrosine kinase domain (TRD), proline rich domain (PRD) and two serine/threonine rich domains [1]. The ROR1 signaling is reported in multiple cases, with different effects: NF-κB activation by Wnt5a binding to ROR-1 FRZ domain [34]; c-SRC phosphorylation by the allosteric interaction between FZD and EGFR [35]; cell growth modulation via ROR1 phosphorylation induced by MET [36]; or cell migration via stimulated/silenced ROR1 [1].

The first functional investigations of ROR1 indicated its involvement in developmental processes after the observation that ROR1 expression plummets in the late stages of embryogenesis of mice, in the head mesenchyme region [37]. Additionally, ROR1-deficient (ROR1−/−) embryos display perinatally lethal defects due to respiratory dysfunction [38]. Later evidence pinpointed that ROR1 expression is concentrated at the growth cones of immature neurons during development [39]. Further studies confirmed the cellular localization of ROR in the plasma membrane with some cases showing nuclear localization. Different isoforms with varying molecular weights were also identified [34, 40–44].

ROR1 functions as a receptor for Wnt5a and other related Wnt proteins, playing an important role in regulating cell migration during embryonic development by interacting with these proteins [11, 45, 46].

WNT/ROR signaling pathway is linked to tumor progression, cell proliferation, and resistance to therapy. WNT/ROR signaling has been linked to the activation of multiple signaling pathways, including MAPK/ERK, STAT3, and NF-kB. The exact mechanisms and interplay between these pathways, as well as the specific functions of ROR1, are not yet completely understood [46–48].

The signal transduction mediated by ROR1 phosphorylation results in the inhibition of proapoptotic pathways and activates downstream pathways that trigger cytoskeletal rearrangements and tumor cell migration, or promote cell proliferation, survival, epithelial to mesenchymal transition, or resistance to therapy.

## Physiological roles of ROR1

In healthy tissues, both ROR1 and ROR2 are important in embryogenesis as receptors for Wnt ligands, while their expression is downregulated in most adult tissues [2, 29, 44, 49]. ROR1 is expressed in immature B cells and tonsillar B cells but is absent in mature B cells, PBMCs, and plasma cells [49–51]. However, the activated T and B cells from healthy donors expressed higher levels of ROR1 mRNA after exposure to ionomycin [52].

ROR1 receptors are cell surface proteins involved in cell-cell interactions, proliferation, survival, differentiation, and even cell metabolism [32]. The gene encoding for ROR1 is located on chromosomal region 1p31.3. Studies on mice have shown that ROR1 is predominantly expressed in developing tissues and plays a significant role in skeletal muscles [53, 54]. Its expression, typically absent in adult tissues, is reactivated in many tumors. In humans, ROR1 exhibits low expression levels in the parathyroid glands, pancreatic islets, adipose tissue, lungs, and gastrointestinal tract, but its expression is increased in tumor cells [22, 30, 34].

After embryogenesis, the expression of ROR1 is downregulated in most adult tissues. However, ROR1 is involved in osteogenesis, acting as a coreceptor for some WNT proteins [53, 55]. Furthermore, studies in mouse models have shown that resistin interacts with the extracellular region of ROR1, inhibiting its tyrosine phosphorylation, which in turn affects ERK1/2 phosphorylation and glucose transporter (GLUT4) [56].

Ho et al. conducted genetic loss of function experiments and showed that loss of ROR1 and ROR2 expression leads to defects in tissue elongation and sympathetic axon innervation that resembled the phenotype of Wnt5a knockout mice. This provides further support that ROR receptors are important mediators of Wnt5a signaling in development. In addition, this study showed that Dvl2 phosphorylation is a key target of Wnt5a-ROR signaling, and neither β-catenin–dependent Wnt signaling, nor c-Jun phosphorylation are involved, providing new insights on the Wnt5a-Ror pathway and its association with noncanonical Wnt signaling [11, 52, 57, 58].

ROR1 is linked to the WNT pathway through its interaction with WNT5a and other WNT proteins. After embryonic development, ROR1 expression is nearly completely reduced. However, in cancer, ROR1 is reactivated, which stimulates tumor cell growth, differentiation, and proliferation through the WNT/ROR1 pathway. In contrast, normal tissues utilize other RTKs to regulate metabolism, cell growth, and survival [41, 59].

## ROR1 in cancer biology

ROR1 is initially synthesized as a104 kDa polypeptide, which, after posttranslational glycosylation and mono-ubiquitination, is expressed in the plasma membrane as a 130 kDa glycoprotein [43].

**Fig. 2:**
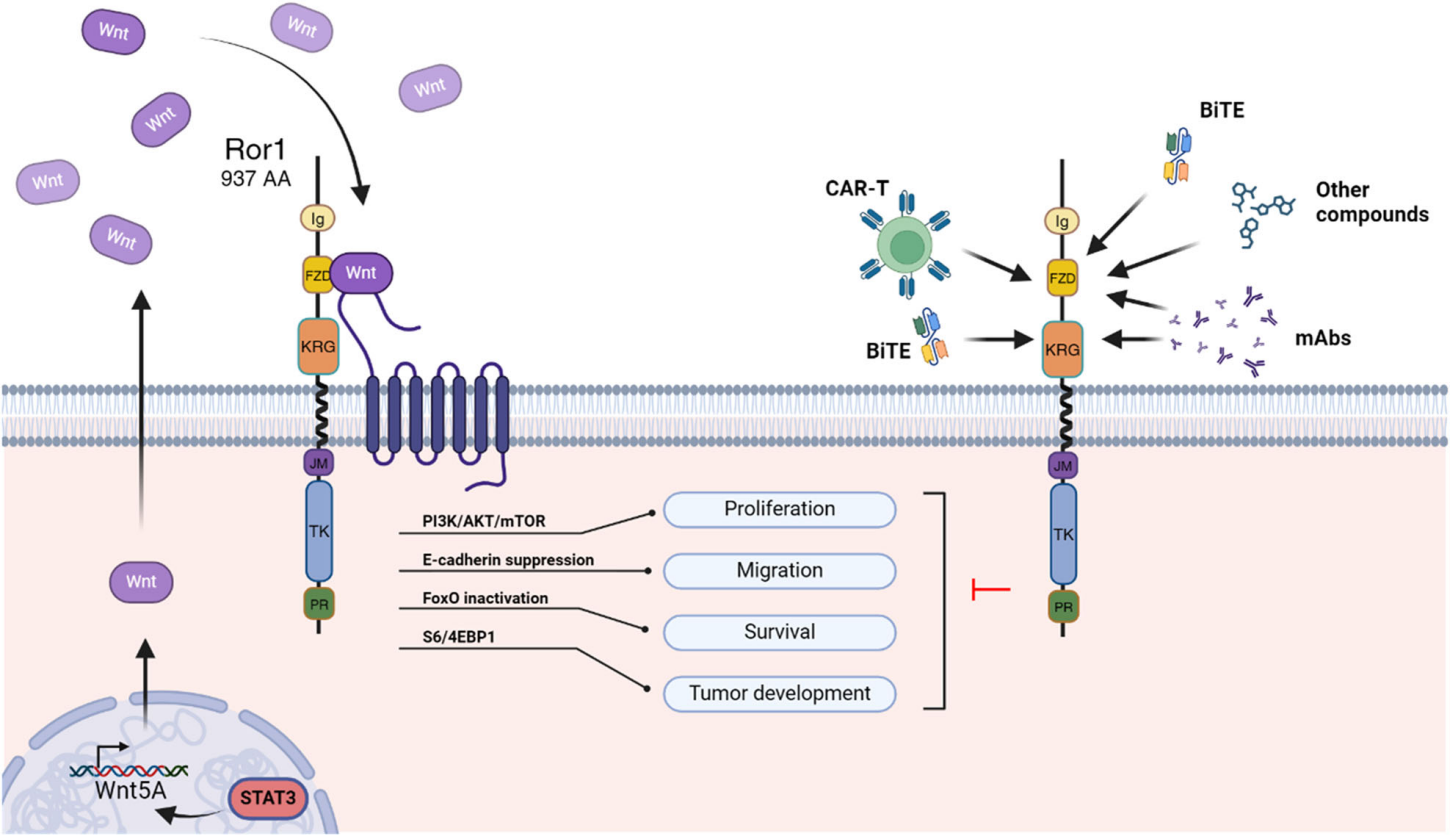
ROR1 signaling and targeted therapies for tumor cell inhibition. The WNT binding to FZD domain triggers downstream signals that support proliferation, stimulate migration and tumor development by inhibiting programed cell death. Targeted therapies, such as CAR T, BiTEs, mAbs and other compounds bind the external subunits of ROR1 and further inhibit critical signaling pathways that control tumor cell survival, proliferation, migration and development (Created with BioRender.com).

STAT3, a key transcription factor, plays a crucial role in regulating ROR1 expression. This regulatory effect is observed specifically in cells that already express ROR1, highlighting the context-dependent nature of this interaction. Interestingly, STAT3 also enhances Wnt5a expression by binding to the WNT5A promoter, creating a complex signaling network (Fig. 2). The significance of STAT3 in this pathway has been further demonstrated through silencing experiments. When STAT3 is silenced, it results in reduced expression of both Wnt5a and ROR1, leading to increased apoptosis in tumor cells. These aspects highlight the importance of STAT3 in maintaining the survival of certain cancer cells through the ROR1-Wnt5a axis [60, 61].

As a result, ROR1 is essential for the crosstalk between WNT signaling and other pathways, particularly PI3K/AKT/mTOR pathway. Activation of ROR1 triggers the phosphorylation of several key proteins, including PI3K, AKT, mTOR and the downstream effectors S6 and 4EBP1. This signaling cascade is related to tumor cell development and proliferation [62–65].

ROR1 expression was evaluated in both hematological malignancies and solid tumors and has been associated with poor outcome and reduced therapeutic responses in several cancers including breast cancer [57, 66], CLL [26, 52, 67, 68], colorectal cancer [69], endometrial cancer [70], lung adenocarcinoma [71], melanoma [63] and ovarian cancer [72, 73].

ROR1 interacts with WNT5A on the cell surface and activates the pathway. Other Wnt proteins can also bind to ROR1, as seen in conditions such as B-cell precursor acute lymphoblastic leukemia (BCP-ALL) and CLL [11].

ROR1’s initial identified role is the activation of Ras homolog family member A (RHOA), Rac family small GTPase (Rac) and cell division cycle 42 (CDC42) in different tumoral tissues [11]. Given its implication in cell survival, ROR1 has become a target for therapeutic strategies, including mAbs, Ab-drug conjugates, BiTEs. Some of these approaches have been evaluated in clinical trials, indicating the potential of targeting ROR1 in cancer treatment [11].

Downstream, the PI3K activation triggers AKT which is controlling the EMT-related genes via E-cadherin suppression, confirming ROR1 involvement in this biological process. Additionally, ROR1 can inactivate FoxO, a transcription factor that promotes apoptosis, suggesting that the crosstalk between these pathways may be important for the survival of tumoral cells regulated by ROR1 expression [35, 74–76]. Recent studies have shown the complex relationship between Wnt5a/ROR1 signaling and other signaling pathways and proteins. Specifically, the crosstalk between Wnt5a/ROR1 signaling and BMI-1 can result in genomic instability and resistance to therapy. On the other hand, the interaction between Wnt5a/ROR1 signaling and YAP/TAZ pathway may enhance cell proliferation and tumor development [77, 78].

Targeted anti-ROR1 therapy is considered a promising strategy for treating cancer because ROR1 is frequently overexpressed on the surface of cancer cells and is associated with tumorigenesis and drug resistance. Inhibiting ROR1 activity could also potentially disrupt the signaling pathways involved in cancer development and progression, leading to a reduction in tumor growth and an improved response to therapy. Furthermore, by specifically targeting ROR1, this strategy may decrease toxicity to normal cells and reduce side effects compared to more generalized therapies However, it is important to note that when CAR T therapies targeting ROR are used, challenges such as immune effector cell-associated neurotoxicity syndrome (ICANS) and cytokine release syndrome (CRS) remain to be effectively managed [79–81].

## ROR1 in hematological malignancies

Since 2001, ROR1 has been identified as a specific marker for CLL [82, 83] with an increasing expression upon CLL progression [51]. CLL patients show ROR1-positive tumor cells in the blood, however, 5% of the reported cases had a very low expression of ROR1 [26]. ROR1 is not an exclusive marker for CLL, it is also expressed in MCL and in about half of the pediatric acute lymphatic leukemia (ALL) cases [49, 84, 85].

Therapeutic compounds were developed after the discovery of ROR1and based on Wnt5a-ROR1 interaction. Cirmtuzumab was developed to compete with Wnt5a and limit proliferation signaling. Furthermore, various other molecules that bind ROR1, including miRNAs, small molecules, monoclonal antibodies, were demonstrated to block proliferation [25, 86–91].

ROR1 requires phosphorylation of its proline rich domain (PRD) to activate downstream signaling cascade, thus the Wnt5a binding induces cell survival in CLL [26, 34, 92]. ROR1 promotes survival in lung cancer cells, and its inhibition decreases the expression of anti-apoptotic proteins like Bcl-2 and Bcl-XL, while increasing the expression of pro-apoptotic proteins, leading to apoptosis [93].

Moreover, stimulation of ROR1 with Wnt5a increased the migratory potential of tumor cells compared to the ROR1 negative cells [26] and similar observations were made on solid cancers, like chemo resistant breast cancer where ROR1 inhibition triggered p53 activity and ABC family of ATP-dependent drug efflux pumps via MAPK/ERK pathway [63, 94–98].

ROR1 can be modulated by miRNAs, which can downregulate ROR1 expression by binding to its mRNA, leading to reduced ROR1 protein levels. In CLL the del (13q) results in the loss of miRNA-15a and miRNA-16-1, leading to increased ROR1 expression and promoting cancer cell survival by overexpressing BCL-2. As a result, the combination of cirmtuzumab with a BCL2 antagonist (venetoclax) show additive or synergistic effects, in vitro [25, 99].

### Therapeutic strategies for targeting ROR1 in hematological malignancies

#### Small molecules

Small molecule inhibitors targeting ROR1 were developed for blocking the activity of ROR1 and disrupting the signaling pathways involved in tumorigenesis and cell proliferation. These small molecules can target both ROR1’s extracellular and intracellular domains and can prevent classical ligand binding. Several small molecules inhibitors targeting ROR1 are currently in preclinical and early clinical development, although their efficacy and safety in humans are still being evaluated.

KAN0441571C, a small molecule with pro-apoptotic effect, is showing promising results in MCL, targeting ROR1 TKD intracellular domain. KAN0441571C was tested alone and in combination with other mechanism of action (MOA) that are currently used in clinical practice (ibrutinib, venetoclax, idelalisib, everolimus and bedamustine) and the results suggest that combinations with small molecules increase apoptosis and inhibit tumor cell survival [100]. Moreover, in the case of CLL ROR1 positive cells that are resistant to ibrutinib, KAN0441571C and venetoclax increased the rate of apoptosis in tumor cells [89, 101]. Similar results were obtained in DLBCL ROR1 positive cells, where KAN0441571C induced apoptosis in tumor cells and in zebra fish models [27].

#### Monoclonal antibodies

Due to the variety of biological interactions, ROR1 represents a target for specific therapies in CLL and other cancers which are reported to express ROR1. Several monoclonal antibody (mAb) therapies showed heterogenous results against ROR1 in CLL, with low levels of cellular toxicity reported in CLL and MCL cell lines treated with anti-ROR1 mAbs [102, 103]. For example, the development of mAbs against FZD and KRD extracellular domains however induced high toxicity in primary CLL samples, and the cytotoxicity was higher than the one induced by rituximab – mAb targeting CD20 [51, 104]. Cui B et al. demonstrated that the use of mAbs against ROR1 reduces metastasis in lungs, assessing the effects in TNBC mouse xenografts [94].

Clinical trials have evaluated the use of cirmtuzumab (NCT02222688; NCT02860676), a monoclonal antibody that specifically targets ROR1. In CLL patients, these trials demonstrated a reduction in the number of tumor cells and cirmtuzumab was found to be safe and effective in targeting ROR1 [105, 106]. Moreover, additional anti-ROR1 antibodies, like UC-961, are also explored [106]. UC-961 binds a distinctive epitope on ROR1 and inhibits its signaling [105].

The effect of cirmtuzumab is currently being is evaluated in a Phase 1 and Phase 2 clinical trial (NCT03088878) for B-CLL, SLL, MCL and marginal zone lymphoma, at different doses. It is being tested in combination with ibrutinib and compared to ibrutinib alone. The study is ongoing and still enrolling patients. Based on the current literature this combination therapy shows potential as a promising treatment option for B-cell lymphoid malignancies [107].

**Fig. 3:**
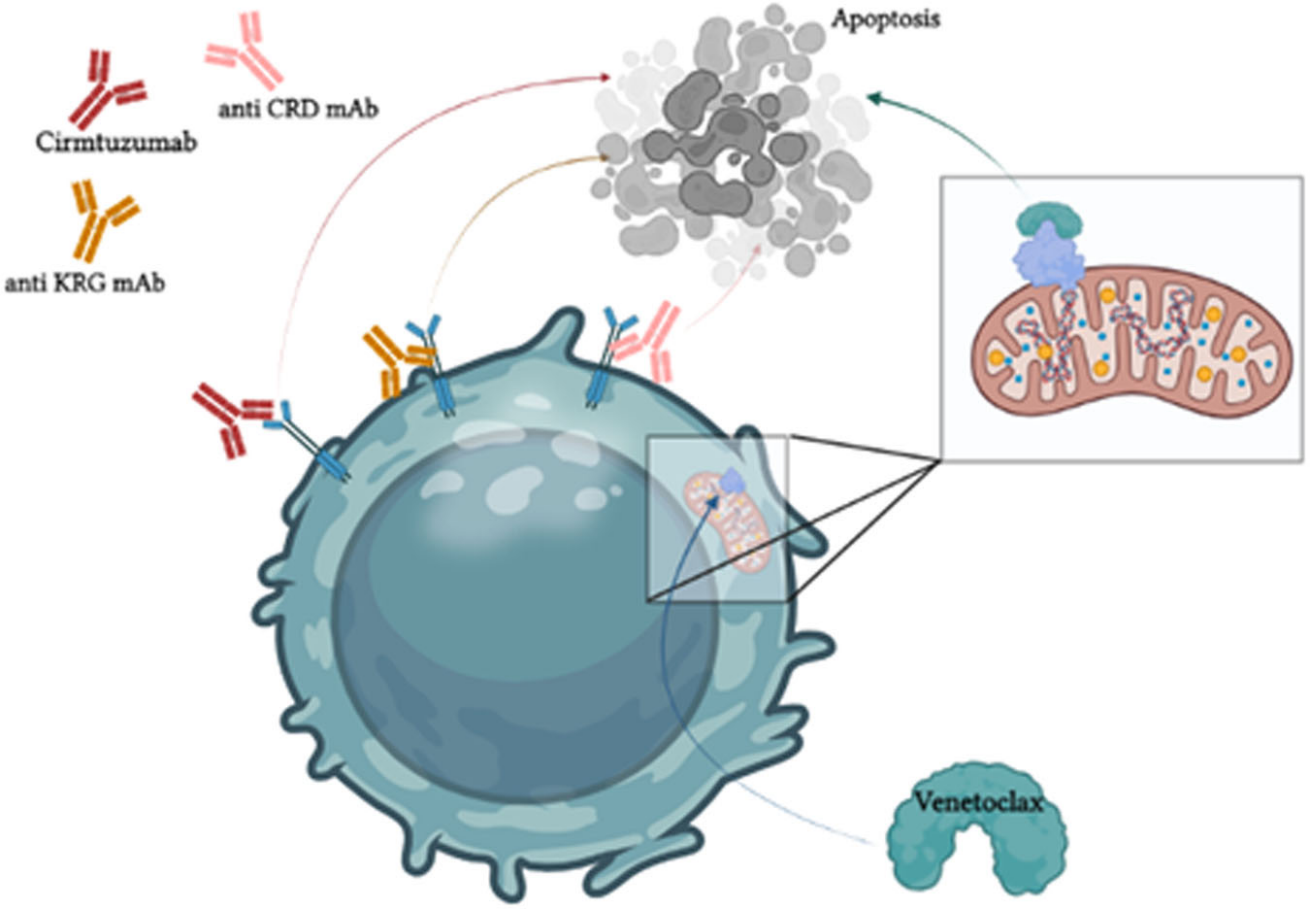
The synergistic effect of venetoclax combined with anti-ROR1 mAbs in CLL. Both therapies lead to an increased apoptosis with a synergistic effect when used as a combine therapy.

The combination of monoclonal antibodies against ROR1 and venetoclax presented synergistic effects against CLL cells [25], making this a potential effective therapeutic approach (Fig. 3) [25].

#### Antibody drug conjugates

Zilovertamab vedotin (ZV), an antibody drug conjugate which is capable to target the extracellular domain of ROR1, conjugated with the anti-microtubule cytotoxin monomethyl auristatin E was evaluated on 32 patients with MCL, CLL, DLBCL, follicular lymphoma, marginal zone lymphoma or Richter transformation lymphoma and showed no unexpected toxicities and antitumor activity [108]. The ZV is evaluated in a Phase 2 clinical trial (NCT04504916) against TNBC, non-TNBC HER2-negative breast cancer, NSCLC, gastric cancer, pancreatic cancer, and platinum-resistant ovarian cancer, with the study still ongoing. ZV is also evaluated in patients with different hematological malignancies, for example, the Phase 1 clinical trial (NCT03833180) which is focused on refractory hematological malignancies including, CLL, DLBCL, MCL and small lymphocytic lymphoma (SLL) [108]. The preliminary data, after 14 months of follow-up, ZV demonstrated a manageable safety profile and promising antitumor activity in patients with DLBCL, MCL, and Richter’s transformation [109].

Another anti-ROR1 antibody drug conjugate, consisting of the huXBR1-402 antibody conjugated with anthracycline derivative (PNU) showed inhibitory effect on ROR1 positive B-cell lymphoma in vivo and MCL in vitro [110, 111].

NCT05279300 is a Phase 1a/b clinical trial involving a novel anti-ROR1 antibody drug conjugate which is tested on advanced solid tumors and lymphomas. The compound is named CS5001, and the results obtained for 49 patients (17 with lymphomas and 32 with solid malignancies) showed that CS5001 is well tolerated and has promising antitumor activity [112].

#### CAR T and BiTEs

Chimeric antigen receptor (CAR) T cell therapy is based on the engineered T cell to target a specific surface antigen and represents a new pillar in oncological therapy. CAR T cell therapy uses isolated T cells which are further genetically modified to target specific antigens. [113–115].

In 2017, Kymriah was approved for pediatric use up to 25 years old B-ALL patients [116]. Yescarta was approved for adult patients with B-cell lymphoma including DLBCL [113], while in 2020, Tecartus was approved for ALL and MCL patients [117].

Anti-ROR1 CAR T cells were designed and tested against CLL and MCL primary cells and induced the lysis of these cells. However, the CAR T therapy targeting ROR1 needs deep evaluation because of its potential toxicity due to the expression of ROR1 in several normal adult tissues, including parathyroid, pancreatic islets, duodenum, regions of the esophagus, stomach and duodenum [29], [49].

BiTEs, are an alternative approach to cancer immunotherapy, distinct from CAR T cell therapy [118]. BiTE structure contains two antibody-derived scFv linked in tandem, one targeting T cell receptor CD3 and the 2nd one targeting a specific molecule [119]. BiTEs are designed to target different specific molecules, such as ROR1 [19].

A panel of rabbit anti-human ROR1 mAbs were generated by phage display. These mAbs have different binding epitopes on ROR1 with high affinity and specificity [103, 120, 121]. Also, they showed increased cytotoxicity against ROR1 malignant cells expressed by CAR T. The CAR constructed from the R12 scFv was evaluated in nonhuman primates, showing no toxicity in normal tissues, and accumulating in locations rich in ROR-1 positive B-cells, such as bone marrow and lymph nodes, and supporting further clinical evaluations [122]. Harnessing the strength of T cells to eradicate tumor cells, Bispecific T cell engagers (BiTEs) are a powerful therapeutical approach and unlike CAR T cells, the BiTEs are administered and manufactured as conventional mAbs [121, 123]. For example, blinatumomab, an CD19/CD3 BiTE was approved by FDA in 2014 for R/R B cell-precursor ALL [124]. While both CAR T and BiTEs can cause adverse effects such as neurological toxicity and cytokine release syndrome, they still show promising results. These approaches represent potential new immunotherapies for targeting ROR1 in hematological malignancies, expanding treatment options for patients with severe diseases and poor outcomes.

### ROR1 as a potential target inCLL therapies

ROR1 is a very promising target for the treatment of CLL, particularly in conjunction with venetoclax treatment, however, some therapeutical approaches are still in early stages of development, many drugs are tested to determine their safety and efficacy and further studies are needed to determine if CAR T cell therapy, small molecules or monoclonal antibodies are a safe and efficient option in CLL management.

ROR1 acts as a receptor for Wnt5a, a protein encoded by WNT5A gene. Wnt5a binds to ROR1 and other Wnt5a receptors and stimulate epithelial to mesenchymal transition (EMT), migration, invasion, and cell growth, moreover it increases both cell growth and chemoresistance via AKT pathway; Wnt5a is also involved in senescence, inflammation and cell proliferation by modulating different biological pathways [125–127].

Drug resistance is a problem in cancer treatment, even in cases of very effective targeting and tumor regression like in the use of venetoclax in CLL, since in the malignancies there might be present rare mutant/variant cells resistant to venetoclax that could expand during treatment. The combination treatment of venetoclax and anti-ROR1 therapy would likely avoid resistance, since it is unlikely that the same tumor cell, hit by two different drugs exploiting two different mechanisms, will be able to survive [25].

According to Peng [128] next-generation ADCs are being investigated, with some of them designed to target ROR1. NBE-002, a next-generation ADC that targets ROR1, is the result of conjugation between huXBR1-402 humanized antibody with anthracycline PNU-159682 and binds to the immunoglobulin domain of human ROR1 and inhibit tumor cell growth [111, 129]. NBE-002 is also included in NCT04441099 phase 1/2 clinical trial [128]. The study consists of a Dose Escalation Cohort (DEC) in Phase 1, followed by expansion cohorts in Phase 2 [130]. On the other hand, VLS-101 (VelosBio101), an ADC with a proteolytically cleavable linker is connected to a tubulin polymerization inhibitor – monomethyl auristatin E [131]. VelosBio101 is based on the UC-961 antibody which binds to ROR1 and block Wnt5a binding to ROR1 [106] and showed limited effect in NCT02222688 clinical study on CLL.

Clinical testing showed a high tolerance of cirmtuzumab in relapsed CLL patients, moreover, the treatment inhibited RhoA and HS1 activation via the inhibition of ROR1 [105, 132]. This effect was also observed for ROR1-positive ovarian cancer cells [71, 105]. HS1 inhibition could reduce the pro-survival effect of Wnta5a ROR1 pathway due to the lack of ROR1-HS1 complex formation [133].

Wnt5a seems to induce the formation of ROR1-DOCK2 complex (dedicator of cytokinesis 2) and induce further activation of Rac1/2 further stimulating proliferation, however, the cascade can be inhibited by the binding of cirmtuzumab to ROR1 [134].

NCT02706392 Phase 1 clinical study, is focused on autologous CAR T cells with a monoclonal antibody R12 for patients with CLL, MCL, ALL, TNBC, and NSCLC. The data shows no unspecific toxicity whereas before the clinical study, these CAR T cells had good response in human tumor xenograft models with ROR1-positive cells [49, 128, 135, 136].

ROR1 seems to induce spontaneous humoral response and T cell response in CLL patients, thus it may represent a good target for immunotherapy [137].

It was demonstrated that CLL patients with high ROR1 expression had shorter median treatment-free survival and overall survival compared to the CLL patients with low ROR1 expression [26]. Furthermore, high levels of ROR1 and an increased signaling downstream to the Wnt5a/ROR1 pathway is associated with venetoclax resistance in CLL [138]. Liu et al. showed that ROR1 creates a loop with heat shock protein 90 (HSP90) leading to a high stabilization of ROR1 and lower the efficacy of Ibrutinib against CLL, however a ROR1 depletion by targeting HSP90 significantly increased Ibrutinib activity against CLL [139].

In another study, Daneshmanesh et al. demonstrated that ROR1 expression is significantly higher in CLL patients with progressive versus non-progressive disease and the ROR1 epitopes were used to generate anti-ROR1 mAbs which seem to induce apoptosis, showing complement-dependent toxicity and antibody-dependent cellular toxicity, as rituximab [104]. Another therapeutical approach could be the use of single-chain Fragment variable (scFv) antibodies, that can target the extracellular domain of ROR; the scFv antibodies showed efficiency against lymphoma and myeloma cell lines, inducing apoptosis and cell death in RPMI8226 plasmacytoma and CLL cell lines [140]. Another compound, KAN0439834, a small molecule inhibitor of ROR1, showed promising results by inhibiting cell proliferation and inducing apoptosis [90].

MiRNA-15a and miRNA-16-1 loss leads to ROR1 and BCL-2 overexpression, thus a combination of venetoclax and antiROR1 therapy (monoclonal antibodies, small molecules and CART cells may decrease the possibility of drug resistance [25]. Other micro RNAs like miRNA-29b induced a downregulation of DNMT1 and DMNT3A modulating DNA methylation, decreased SP1 and increased expression of p21, both in vitro and in vivo; thus, a cell cycle arrest was induced and increased the survival of Eu-TCL1 mice [141].

### MCL therapies - ROR1 as a potential therapeutical target in MCL

Mantle cell lymphoma (MCL) is a B-cell non-Hodgkin lymphoma, a rare disease with clinically heterogenicity [142]. MCL is characterized by the chromosomal translocation (11;14) which leads to Cyclin D overexpression and overstimulated proliferation. The therapeutical approach is dependent on patient-specific factors, such as age, underlying comorbidities, and overall performance status. While the young patients are eligible for transplant and common regimens with rituximab/dexamethasone/cytarabine/cisplatin or vincristine/prednisone, for unfit patients, bendamustine/rituximab can be used as a less toxic alternative [143].

High-dose chemotherapy and CD19 CAR T cell therapy is one therapeutical option for MCL. Clinical trial NCT02614066 is evaluating the anti-CD19 CAR T cell therapy in R/R B-cell ALL and expecting promising results [144, 145]. Currently, for MCL, Brexucabtagene autoleucel is approved as a CAR T cell therapy, with a very good overall survival, while Lisocabtagene maraleucel is in phase one clinical trial and shows promising results [146].

ROR1 is highly expressed in MCL and other B-cell malignancies such as Burkitt’s lymphoma and CLL, as previously described. Moreover, by silencing the ROR1 gene, the effect is reversed by reducing cell growth and increasing cell death [50, 60, 62, 147]. This receptor is also present in DLBCL or marginal zone lymphoma (MZL), although functional characterization of MZL is limited compared to other B-cell malignancies. [16, 148].

Given the rarity of MCL, clinicians must address and identify the best therapeutical approach based on the physiological and clinical aspects of each patient.

Currently, several small molecules are used against specific targets. Burton’s tyrosine kinase (BTK) is one of the targets due to its implications in proliferation and survival via ERK, PI3K and NF-kB, therefore, its inhibition may stimulate apoptosis and inhibit cell proliferation [149]. Ibrutinib is a first-class inhibitor of BTK, it binds to the BTK and irreversibly inhibits the signaling. Furthermore, Ibrutinib inhibits IL2 inducible T cell kinase and EGFR, and in a relapse/refractory MCL pivotal phase 2 study, Ibrutinib demonstrated Overall response rate of 67% [150] and showed promising results in relapse MCL phase III MCL3001 trial NCT0164021 with an improved progression free survival [151]. Zanubrutinib, another BTK inhibitor, which binds to the receptor and irreversibly inhibits the downstream pathway, showed impressive results in R/R MCL on two phase two studies [152, 153].

In MCL therapy, BCL2 inhibitors are also used due to the overexpressed BCL2. For this reason, Venetoclax is used as a highly selective BCL2 inhibitor which demonstrates promising results against RR B cell NHL patients which also include MCL patients [154].

Bortezomib, the only approved proteasome inhibitor, with single-agent activity in RR MCL, is validated and used to inhibit MCL cell proliferation via inhibition of NF-kB signaling [143, 155, 156]. A less toxic alternative for Bortezomib, is Carfilzomib, but with poor outcome compared to Bortezomib. [143, 157–159] Lenalidomide, an immunomodulatory agent, showed good antitumor effect in MCL. However, as a single-agent therapy in RR-NHL, the results were not satisfying. Fifty-seven patients with RR MCL were included in the study. The ORR was only 35%, and the median PFS was 8.8 months [160, 161]. Antibody-drug conjugates (ADC) can represent an alternative MCL therapy. By targeting CD79B, CD22, or CD37 [162], the ADCs induce apoptosis and inhibit cell proliferation [163–165].

Significant improvements were observed in the overall survival of B cell NHL after rituximab, which targets CD20, was approved for clinical use [166]. However, other specific markers like CD37, CD74, or ROR1, that are expressed in MCL can be targeted by antibodies [167, 168].

ROR1, a biomarker for both CLL and MCL, is activated by Wnt5a leading to an increased cell proliferation rate. The most notable antibody that targets ROR1 and blocks Wnt5a binding is Cirmtuzumab, which shows good outcome in ibrutinib resistant MCL and RR CLL. and(NCT03088878) involving the combination of cirmtuzumab and ibrutinib are ongoing [133].

The interaction between ROR1 and CD19 forms a complex that promotes MCL cell growth. Therefore, consequent binding of other receptors or BTK may not be enough to eliminate tumor cells as they can overcome the effectiveness of the therapy [28]. Based on this effect, several molecules that target ROR1 were tested, some of them working efficiently, but presenting side effects. Some examples include Brexucabtagene autoleucel, the first CAR T approved in R/R MCL with high efficacy, or Cirmtuzumab which shows good anti-tumor activity, but where resistance to therapy was reported. To overcome this resistance and potential side effects, a combination of Cirmtuzumab and monomethyl auristatin E, called VLS-101, was designed to target ROR1 and inhibit tumor cells [169]. The efficacy was demonstrated in vivo, on PDX (Patient Derived Xenograft) models of patients with resistance to ibrutinib and CAR T cells therapy [170].

CAR T cells therapy and the use of BiTEs could revolutionize the treatment in hematological malignancies [171]. Currently, in the ZUMA-2 trial (NCT02601313), axicabtagene-ciloleucel was tested in R/R MCL [172–174] and the results indicated a remission in the majority of R/R MCL patients. Blinatumomab, Mosunetuzumab, REGN1979 and GEN3013 are four BiTEs that are currently being tested in clinical trials for R/R B cell NHL, including the MCL subtype [175, 176]. Blinatumomab is being verified in combination with other therapies. On the other hand, Mosunetuzumab shows less toxicity that CAR T cell therapy and Blinatumomab therapy [143].

### DLBCL therapies and ROR1 approaches

Diffuse large B-cell lymphoma (DLBCL) is the most common type of non-Hodgkin Lymphoma, with high heterogenicity and diverse clinical symptoms, variable prognosis, and response to treatments [177]. Common therapies include R-CHOP chemotherapy and rituximab. However, 30% of the patients become refractory to initial treatment and relapse after standard therapies [178]. Due to the high relapse/refractory cases, the identification of novel targets and therapeutical approaches are essential for disease management.

By targeting ROR1, tumor cell growth could be inhibited as ROR1 possesses properties typical of a tumor-associated antigen, and multiple studies reported a relationship between ROR1 and human cancer cells [52, 57, 102, 179].

The expression of ROR1 is associated with poor outcome in DLBCL and other several malignancies due to its importance in tumor cell survival, migration and metabolism. ROR1 expression is more frequently observed in primary refractory DLBCL, Richter’s syndrome and transformed follicular lymphoma, while its expression is lower in relapsed DLBCL [27]. Mao et al. reported that knocking down ROR1 could inhibit the growth of DLBCL cells both in vitro and in vivo, highlighting ROR1 as a potential target for DLBCL therapies [177].

### MM therapies and ROR1 approaches

Also known as plasma cell myeloma, multiple myeloma (MM) is characterized by the uncontrolled growth of clonal plasma cells (PCs) which are capable of producing IgG, IgA and IgD [180]. The plasma cells proliferate in the bone marrow without many reported extramedullary involvements at diagnosis. The increased secretion of monoclonal immunoglobulins will afterward lead to distant organ damage [180, 181].

The accumulation of mutations, epigenetic alterations, or loss of chromosomes in PCs over time can lead to a malignant phenotype, resulting in myeloma [180, 182, 183]. MM progression has three stages and starts from the monoclonal gammopathy of undetermined significance (MGUS) with less than 3 g/dL serum M-protein, less than 10% bone marrow plasma cells (BMPC) and without end-organ damage. Then, the progression leads to smoldering multiple myeloma (SMM) characterized by an increased serum and urinary M-protein, BMPC between 10 and 60% and no amyloidosis or end-organ damage. The last stage of MM is represented by the active MM where more than one focal lesion can be detected by MRI, more than 10% BMPC with evidence of end-organ damage, or more than 60% BMPC without end-organ damage, along with a serum-free light chain ratio (FLC) higher than 100 [123, 180, 184, 185].

In the last 50 years, the pre-clinical and clinical research led to the discovery of many therapies which increased the 5-year survival rate from 20% to more than 60% of the patients. This was achieved with the introduction of proteasome inhibitors (PIs), anti-CD38 antibodies and immunomodulatory agents (IMIDs) [186–189]. Still, many therapeutical challenges remain unsolved, as is the case of the tumor microenvironment, extracellular matrix, cell-to-cell interactions and the potential biological pathways modulation [190–192].

Current European and US protocols, as well as real life-and clinical practice, show multiple standards of care regimens including immunomodulatory drugs like thalidomide or lenalidomide, monoclonal antibodies like daratumumab, ADCs, proteasome inhibitors, histone deacetylase inhibitors, and selective inhibitors of nuclear export like Selinexor [193–195]. In some cases, patients with MM have a poor outcome after different lines of treatments and develop organ damage [181, 196] thus is important to develop new products that can specifically target tumor cells, by binding to different receptors, like ROR1.

As ROR1 could be targeted in solid tumors and hematological diseases [19, 63, 69, 197] it could represent a target for oncological conditions as well as for MM. However, Receptor tyrosine kinase like orphan receptor 2 (ROR2) could be another attractive target for antitumor therapies, with promising results already published [198, 199].

### ROR1—a target in AML

Several studies have reported that ROR1 could also be expressed in AML. Balaian et al. reported a 35% positivity of ROR1 in AML cells from 179 patients. Moreover, their research group demonstrated that the response to anti-ROR1 mAb was significant in about half of the samples [200]. ROR1 was found to be expressed on several AML cell lines such as THP1, MV4-11 and NB4, indicating that ROR1 therapy could be effective in AML [201].

In the case of B-ALL, the expression of ROR1 is related to several chromosomal translocations and mutations which may lead to aggressiveness of specific B-ALL subtypes [50]. The presence of ROR1 was reported on MCL, DLBLC, and several solid tumors. However, the importance of ROR1 was not recognized until 2008 when its expression was related to CLL and it was demonstrated that ROR1 plays a significant role in prognostication [34]. Evidence for the involvement of ROR1 in malignancies is based on its expression on tumor cells. CLL is one of the most notable examples of ROR1 positivity, especially when considered in the context of physiologically ROR1-negative mature B cells [34, 50].

It was reported that ROR1 is involved in the development of many solid and hematological tumor cells (Table 1). Moreover, in the case of CLL it was demonstrated that ROR1 is responsible for resistance to therapy via the expression of genes involved in resistance to cytotoxic drugs [138].

**Table 1:** ROR1 targeted therapies in hematological malignancies.

Disease	Anti-ROR1 therapy	Effect	Clinical Status	Ref.
AML	anti-ROR1 mAb (UC99961)	Dose-dependent inhibition of colony formation in ROR1 positive cells.	Preclinical	[200]
CLL	Small molecule inhibitor KAN0441571C	Induces apoptosis of CLL cells	Preclinical	[101, 202]
CLL	Small molecule inhibitor KAN0439834	Induces apoptosis of CLL cells	Preclinical	[90]
CLL	mAbs targeting ROR1’s CRD and KNG domains	Induces complement-dependent cytotoxicity (CDC) and antibody-dependent cellular toxicity	Preclinical	[104]
CLL	UC-961 (Cirmtuzumab)	Inhibits ROR1 signaling	Phase I Clinical Trial	[105]
DLBCL	Small molecule inhibitor KAN0441571C	Induces apoptosis of DLBCL cells	Preclinical	[27]
MCL	Small molecule inhibitor KAN0441571C	Induces apoptosis of MCL cells	Preclinical	[89]
MCL	mAb – ROR1-immunotoxin BT-1 and fragments of 2A2-IgG	Induces apoptosis of MCL cells	Preclinical	[102]
MCL and DLBCL	Zilovertamab vedotin (ZV)	Inhibits ROR1 signaling	Phase 1 Clinical Trial	[108]
MCL	Antibody-drug conjugate huXBR1-402-G5-PNU	Inhibits ROR1+ cells proliferation	Preclinical	[111]
MCL	BiTE – ROR1 and CD3	Induces cell death by cell lysis	Preclinical	[121]
MCL and DLBCL	Antibody drug conjugate CS5001	Inhibits ROR1+ cells proliferation	Phase 1a/b Clinical Trial	[112]

## Conclusions

The important fact that ROR1 is on the cell surface and is an embryonal antigen not expressed in normal somatic cells strongly supports the use of anti-ROR1 therapies in cancer. While it is not yet conclusively proven, current evidence suggests that overexpression of ROR1 could be a co-driver of malignant transformation. Inhibition of ROR1 with small molecule drugs or monoclonal antibodies may decrease receptor activity and potentially disrupt critical signaling pathways.

At the same time, since ROR1 is expressed on the surface of cancer cells, monoclonal antibodies against ROR1, that induce cytotoxicity, could represent a therapeutical approach. Recent studies have shown that combining cirmtuzumab with venetoclax, a BCL2 inhibitor, have shown to be synergistic in inducing apoptosis of CLL cells in vitro. Similarly, CAR T cells could be exploited to inhibit the proliferation of ROR1 positive malignant cells.

Moreover, targeting ROR1 using different drug formulations represents a promising strategy for overcoming drug resistance in cancer treatment. This is possible because the receptor can interact with different molecules, such as mAbs, small molecule inhibitors, BiTEs, or antibody-drug conjugates, through multiple mechanisms. Targeting different domains of the receptor or combining ROR1 inhibitors with other therapies, including chemotherapy or immune checkpoint inhibitors, could reduce the risk of resistance to therapy.

While CAR T cell therapy has potential as targeted therapy, the combinatorial therapies involving CAR T and single-agent therapies could significantly increase treatment efficacy. However, the significant potential of combined therapies should consider the risk of toxicity, resistance development, and effects on the tumor microenvironment.

One potential enhancer for anti-ROR1 therapies is the use of miRNAs, which can target specific regions of mRNA that encode ROR1, leading to its downregulation, or miRNAs influence related signaling pathways. Combining miRNA therapy combined with anti-ROR1 agents may have synergistic effects and may enhance the immune response.

Given ROR1’s role in promoting tumor development, anti-ROR1 therapies represent a promising approach in hematological malignancies. By targeting ROR1, these therapies can disrupt specific oncogenic signaling pathways that cancer cells depend on for survival, proliferation, and metastasis, potentially offering durable remissions and improved outcomes for patients with aggressive malignancies.
